# AHR mediates the aflatoxin B1 toxicity associated with hepatocellular carcinoma

**DOI:** 10.1038/s41392-021-00713-1

**Published:** 2021-08-09

**Authors:** Qing Zhu, Yarui Ma, Junbo Liang, Zhewen Wei, Mo Li, Ying Zhang, Mei Liu, Huan He, Chunfeng Qu, Jianqiang Cai, Xiaobing Wang, Yixin Zeng, Yuchen Jiao

**Affiliations:** 1 State Key Laboratory of Molecular Oncology, National Cancer Center/National Clinical Research Center for Cancer/Cancer Hospital, Chinese Academy of Medical Sciences and Peking Union Medical College, Beijing, China; 2 State Key Laboratory of Medical Molecular Biology, Institute of Basic Medical Sciences Chinese Academy of Medical Sciences, School of Basic Medicine Peking Union Medical College, Beijing, China; 3 Department of Hepatobiliary Surgery, National Cancer Center/National Clinical Research Center for Cancer/Cancer Hospital, Chinese Academy of Medical Sciences and Peking Union Medical College, Beijing, China; 4 Key Laboratory of Gene Editing Screening and R&D of Digestive System Tumor Drugs, Chinese Academy of Medical Sciences, Peking Union Medical College, Beijing, China; 5 State Key Laboratory of Oncology in South China, Collaborative Innovation Center for Cancer Medicine, Sun Yat-sen University Cancer Center, Guangzhou, China; 6 Department of Clinical Laboratory, National Cancer Center/National Clinical Research Center for Cancer/Cancer Hospital, Chinese Academy of Medical Sciences and Peking Union Medical College, Beijing, China

**Keywords:** Gastrointestinal cancer, Gastrointestinal cancer

## Abstract

Aflatoxin exposure is a crucial factor in promoting the development of primary hepatocellular carcinoma (HCC) in individuals infected with the hepatitis virus. However, the molecular pathways leading to its bioactivation and subsequent toxicity in hepatocytes have not been well-defined. Here, we carried out a genome-wide CRISPR-Cas9 genetic screen to identify aflatoxin B1 (AFB1) targets. Among the most significant hits was the aryl hydrocarbon receptor (AHR), a ligand-binding transcription factor regulating cell metabolism, differentiation, and immunity. *AHR*-deficient cells tolerated high concentrations of AFB1, in which AFB1 adduct formation was significantly decreased. AFB1 triggered AHR nuclear translocation by directly binding to its N-terminus. Furthermore, AHR mediated the expression of P450 induced by AFB1. AHR expression was also elevated in primary tumor sections obtained from AFB1-HCC patients, which paralleled the upregulation of PD-L1, a clinically relevant immune regulator. Finally, anti-PD-L1 therapy exhibited greater efficacy in HCC xenografts derived from cells with ectopic expression of AHR. These results demonstrated that AHR was required for the AFB1 toxicity associated with HCC, and implicate the immunosuppressive regimen of anti-PD-L1 as a therapeutic option for the treatment of AFB1-associated HCCs.

## Introduction

Among the common malignancies around the world, primary liver cancer gives rise to the fourth-highest number of malignant tumors in China^1^. Surgery is currently the main standard-of-care strategy for the treatment of the disease, although the 5-year recurrence rate and metastasis remain high^2,3^. Chronic infection of hepatitis B virus (HBV) and hepatitis C virus (HCV), as well as aflatoxin exposure in the diet account for the major causative factors of liver cancer^4^. Due to the contamination of the food supply with aflatoxin, particularly in South China, the local population is at an increased risk for the development of hepatocellular carcinoma (HCC)^5–7^. Therefore, clinical and basic research studies performed on aflatoxin-related liver cancer are crucial for the disease prevention, clinical diagnosis, and therapy of liver cancer in China^8^.

Derived from *aspergillus* fungi, aflatoxin B1 (AFB1) is highly carcinogenic. It significantly suppresses the immune response, thereby increasing the risk rate of developing cirrhosis and HCC in chronic HBV carriers^9,10^. The coincidence of HBV infection generally accelerates the development of HCC^11^. AFB1 binds with DNA covalently to form AFB1-N7-guanine, the key adduct responsible for the genotoxicity of AFB1. Members of the cytochrome P450 oxidase family, including CYP1A2, CYP3A4, and CYP2A6, are the main enzymes for catalyzing the generation of AFB1-N7-guanine^12–15^. In contrast, little research about the uptake and transport of AFB1 in targeted cells.

The cytochrome P450 isoenzymes are terminal oxidases in the mixed-function oxidase system of the endoplasmic reticulum that act as a pivotal role in the detoxification of exogenous substances, homeostasis, and cellular metabolism^16^. It is now clear that human cytochrome enzymes are related to the metabolism of multiple exogenous substances, including drugs, alcohol, chemicals, antioxidants, organic solvents, dyes, anesthetics and environmental pollutants, and the carcinogenic produced metabolites^17^. One mechanism for protection from external pollutants, toxins, and pathogens is the aryl hydrocarbon receptor (AHR), a receptor protein which is mainly expressed in various barrier positions in the body^18^. Research has been demonstrated AHR could bind with a series of metabolites derived from endogenous or exogenous resources, such as tryptophan metabolites, microbial-derived factors and dietary components, and mediate their actions^19^. The AHR/P450 pathway thus plays a crucial role in maintaining physiological homeostasis.

In this work, we systematically searched for the functional elements required for AFB1-induced cell death and identified AHR as a new and important factor mediating AFB1-related toxicity. This result highlights the critical role of AFB1 uptake in the generation of toxicity and downstream carcinogenic effects in HCC, thereby providing a new avenue for the medical treatment and prevention of aflatoxin-induced liver cancer.

## Results

### AHR is requisite for the cellular toxicity of AFB1

**Fig. 1:**
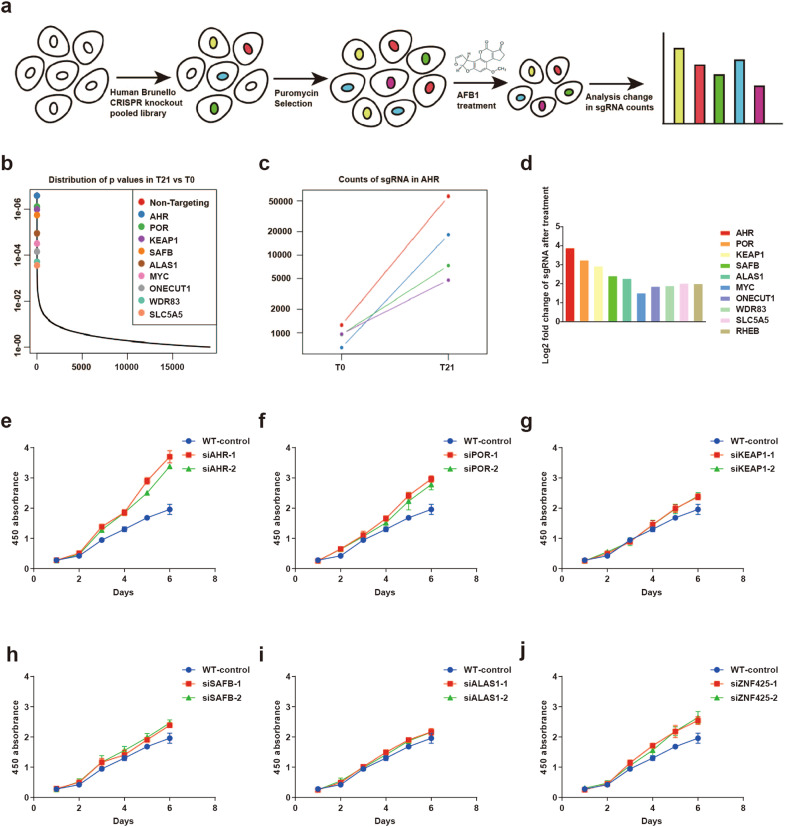
Genome-wide CRISPR-Cas9 screen identifies genes essential for AFB1-induced cell death. **a** Schematic of CRISPR-Cas9 function screens. **b** Genes significantly enriched after six rounds of AFB1 treatment were identified through analysis of sequencing results in the MAGeCK program. **c** Change in sgRNA counts after AFB1 treatment relative to untreated cells. **d** Fold change of the sgRNA targeting candidate genes (only the top ten genes are shown). **e–j** Cell viability measured with the CCK8 assay for PLC/PFR/5 cells in 20 µM AFB1 with knockdown of **e** *AHR*, **f** *POR*, **g** *KEAP1*, **h** *SAFB*, **i** *ALAS1*, and **j** *ZNF425*

To reveal the functional network underlying AFB1-induced cell toxicity, we introduced a genome-wide CRISPR-Cas9 library into PLC/PRF/5 cells. AFB1 was maintained at a concentration of 8 µM in the culture by refreshing the compound once every 48 h for six times. Around 10% of the cells survived each round of selection, and the total surviving cells after the six cycles were collected for the final sequencing (Fig. 1a). Among the top hits identified were *POR* and *CYP1A1* (overrepresented 9.524 and 3.647 fold in the surviving cell population), encoding two proteins involved in the cytochrome P450 metabolism, as well as *AHR* (overrepresented 14.807 fold in the surviving cell population), encoding a ligand-activated helix-loop-helix transcription factor (Fig. 1b–d), (Supplementary Table S1). Cytochrome-P450 enzymes are responsible for generating the reactive intermediate AFB1-8, 9 epoxide (AFBO) that primarily gives rise to AFB1 hepatocarcinogenic genotoxicity^20^. The identification of this family of enzymes, therefore, validates our approach with the genetic screen. More importantly, previous work revealed that AHR mediates the actions of some non-genotoxic carcinogens that are metabolized through the cytochrome P450-related metabolic pathway, raising the interesting possibility that AHR may function downstream of the P450 enzymes as a key mediator for the cellular toxicity of AFB1.

A selection of top genes in the screen were targeted individually in PLC/PRF/5 cells via siRNA-mediated knockdown (KD) to validate their role in AFB1 cellular toxicity. Two independent siRNAs were included to monitor potential off-target effects, and their efficiency in suppressing gene expression was confirmed by RT-PCR (Supplementary Fig. S1a–f). Cell survival in the presence of AFB1 was the highest in cells with KD of *AHR*, and less with KD of *POR* and *ZNF452*. However, the remaining targeted RNA is yielded relatively moderate changes in cell viability (Fig. 1e–j). In addition, KD of *AHR* did not affect cell growth in the absence of AFB1 (Supplementary Fig. S2a, b). The overall results indicated that the function of AHR was required for AFB1-induced cell death, which was confirmed in HuH7 hepatoma cells (Supplementary Fig. S1g–l).

**Fig. 2:**
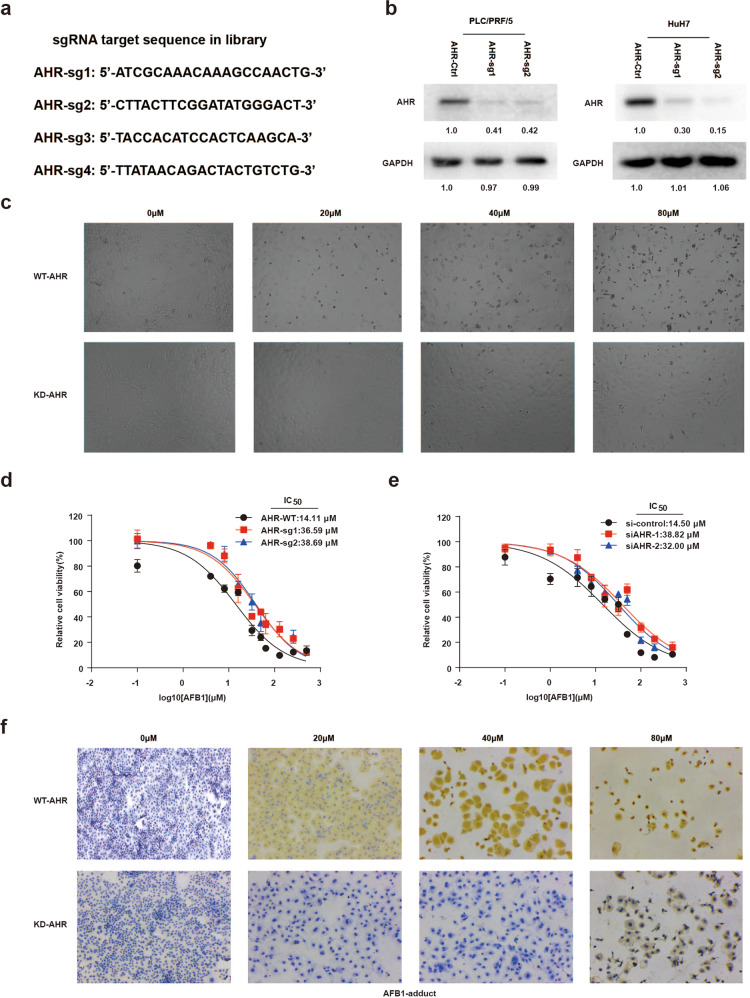
*AHR* deficiency increases the resistance of liver cancer cells to AFB1 treatment. **a** *AHR* sgRNA target sequence in the genome. **b** Western blot to validate *AHR* KD in PLC/PRF/5 and HuH7 cells. **c** Representative images of PLC/PRF/5 cells supplemented in medium containing different concentrations of AFB1 for 48 h. The images were acquired with a LEICA inverted microscope (DMI 4000B). **d**, **e** IC50 of AFB1 in *AHR* WT and *AHR* KD PLC/PRF/5 cells incubated with AFB1 for 48 h assessed with the CCK8 assay. *AHR* knockdown was achieved using CRISPR sgRNA technology in (**d**) and siRNA in (**e**). **f** Immunostaining for AFB1 adducts in *AHR* WT and *AHR* KD PLC/PRF/5 cells

We then focused on understanding AHR’s role in AFB1-related pathogenesis. Both PLC/PRF/5 and HuH7 hepatoma cells were transduced with two independent sgRNAs optimized based on the results of the genetic screen (Fig. 2a). AHR protein was decreased in the independent KD cell lines derived after the puromycin selection, confirming that the *AHR* locus had been disrupted successfully (Fig. 2b). *AHR* KD cells survived in AFB1 concentrations of up to 80 µM whereas the parental cell lines had already become detached and started to die at the AFB1 concentration of 20 µM (Fig. 2c). AFB1 exhibited even stronger toxic effects in liver cancer cells overexpressing *AHR* liver cancer cells (OE AHR; Supplementary Fig. S2c). There was also a significant shift in AFB1 IC_50_ when we compared the KD cells with the parental cell lines. Specifically, the IC_50_ shifted from 14.11 µM in the wild-type (WT) PLC/PRF/5 cells to 36.59 µM in the *AHR*-sg1 KD PLC/PRF/5 cells, and from 20.09 to 75.91 µM in WT and KD HuH7 cells (Fig. 2d and Supplementary Fig. S2d). These results also were confirmed with the RNAi-mediated KD experiments (Fig. 2e and Supplementary Fig. S2e).

The formation of AFB1 adducts is a critical step in the liver cancer progression associated with aflatoxin. Immunostaining revealed a significant reduction in the level of AFB1 adducts in AHR KD cells as opposed to the WT PLC/PRF/5 cells (Fig. 2f), suggesting a functional link between AHR and the metabolism of AFB1 in hepatocyte-derived cancer cells. Finally, because AFB1 exposure has been regarded as an important factor in inducing malignant transformation in the early stages of the development of HCC^21^, we performed colony-forming and CCK8 assays on L-O2 normal liver cells with AHR KD or overexpression. The increase in colonies in KD AHR L-O2 cells demonstrated that AHR deficiency enhanced the malignant transformation ability of normal liver cells under AFB1 treatment (Supplementary Fig. S2f, g). Overall, AHR was critical to the mode of action of AFB1 in these liver cell lines.

### AFB1 induces the accumulation of LCFAs that is mediated by AHR

**Fig. 3:**
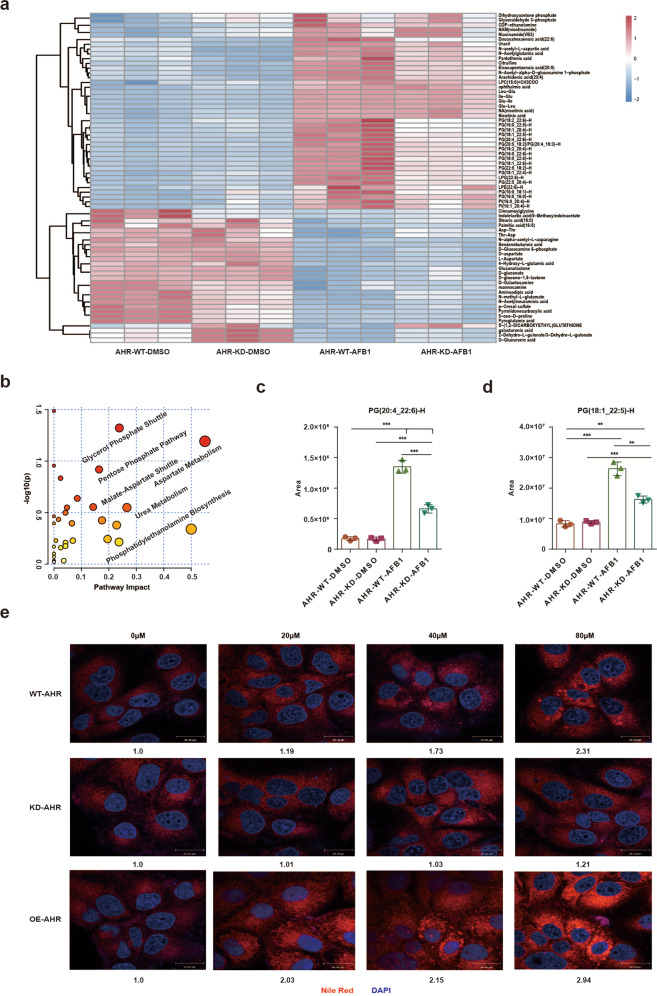
AFB1 increases the accumulation of long chain fatty acids. **a** The heatmap of regulated metabolites extracted from the *AHR*-WT-DMSO, *AHR*-KD-DMSO, *AHR*-WT-AFB1, and *AHR*-KD-AFB1 group in the negative ion mode. **b** Pathway enrichment analysis of regulated metabolites of the four groups in the negative ion mode. **c**, **d** The level of **c** PG(20:4_22:6)-H and **d** PG(18:1_22:5)-H was detected by LC–MS/MS. The results were expressed as mean ± SD. Data among multiple groups were analyzed using two-way ANOVA, *p < 0.05, **p < 0.01, and ***p < 0.001. **e** Representative fluorescence images of cells stained with Nile Red to detect LCFAs (600X). Nuclei are stained with DAPI (blue)

The AHR-P450 pathway is involved in lipid production as well as the metabolism of xenobiotic substances^22,23^. We therefore systematically investigated the metabolic changes caused by AFB1 and mediated by the AHR-P450 pathway. *AHR*-WT and *AHR*-KD PLC/PRF/5 cells were analyzed in pairs to collect the data for nontargeted metabolomics. Both positive and negative ionization LC-MS modes were included to achieve more comprehensive metabolome coverage. The differential metabolites identified are summarized in the heatmaps as shown in Fig. 3a, Supplementary Fig. S3a, and listed in Supplementary Tables S2, 3. The data analysis was performed with MetaboAnalyst 4.0. revealed that the metabolites derived from metabolic pathways involved in the glycerol phosphate shuttle, aspartate metabolism, and pentose phosphate pathway were enriched in the negative ion mode (Fig. 3b), whereas the metabolites derived from glycerophospholipid metabolism, pyrimidine metabolism, and arginine biosynthesis pathways were enriched in the positive ion mode (Supplementary Fig. S3b). Interestingly, the long chain fatty acids (LCFAs) constituted the metabolites that were upregulated by AFB1 in WT cells compared with AHR-KD cells and were represented by PG(20:4_22:6)-H, PG(18:1_22:5)-H, PG(18:1_20:4)-H, and PG(16:0_22:5)-H (Fig. 3c, d and Supplementary Fig. S3c, d).

The accumulation of LCFAs was also monitored in cells during AFB1 exposure (Fig. 3e). The LCFA aggregates began to form at the concentration of 20 µM, and the scale of aggregation appeared in a concentration dependence manner in WT PLC/PRF/5 cells. In contrast, AHR KD cells were significantly less sensitive to AFB1 while AHR OE cells were significantly more sensitive to AFB1 in triggering the accumulation of LCFAs, which is consistent with the metabolomic changes induced by AFB1. LCFAs have also been shown to accumulate during necroptosis and to decrease cell proliferation^24^. We, therefore, measured the expression of necroptosis markers, including MLKL, RIPK1, and RIPK3 under AFB1 treatment, and found that AFB1 treatment-induced their expression (Supplementary Fig. S3e). The overall results indicate that AHR mediates the accumulation of LCFAs specifically induced by AFB1.

### AHR mediates the transcriptional shift induced by AFB1

**Fig. 4:**
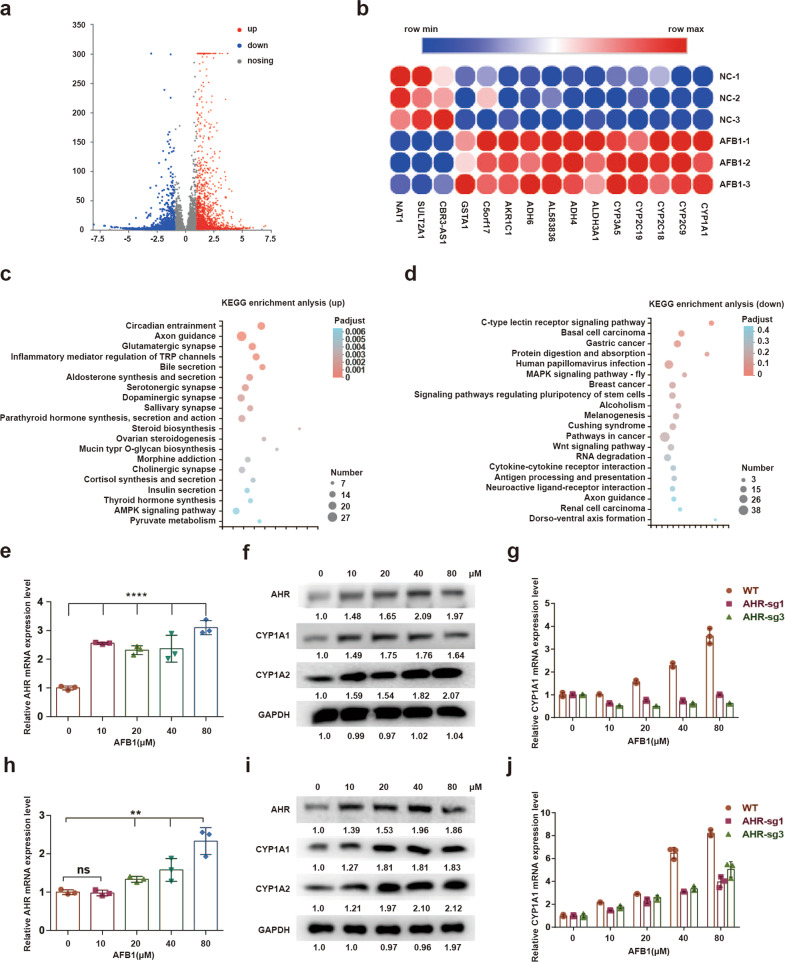
AFB1 activates *AHR*-dependent gene expression in hepatocellular carcinoma cell lines. **a** Volcano plot showing differentially expressed genes upon AFB1 treatment. Blue dots represent downregulated genes (FDR ≤0.05, fold change <0.5) and red dots indicated upregulated genes (FDR ≤0.05, fold change >2). **b** The heatmap of differentially abundant genes was obtained from the NC (PLC/PRF/5 cells treated with DMSO) and AFB1 (PLC/PRF/5 cells treated with 20 µM AFB1) treated groups. **c** Significantly upregulated KEGG pathways with AFB1 treatment. **d** Significantly downregulated KEGG pathways with AFB1 treatment. **e** qPCR to determine *AHR* mRNA expression levels treated with AFB1 in PLC/PRF/5 cells. **f** Western blot analysis for AHR, CYP1A1, and CYP1A2 in PLC/PRF/5 cells. **g** qPCR to determine *CYP1A1* expression levels in *AHR* WT and *AHR* KD PLC/PRF/5 cells treated with AFB1. **h** qPCR to determine *AHR* mRNA expression levels in HuH7 cells treated with AFB1. **i** Western blot analysis for AHR, CYP1A1, and CYP1A2 in HuH7 cells. **j** qPCR to determine expression levels of *CYP1A1* in *AHR* WT and *AHR* KD HuH7 cells treated with different concentrations of AFB1

We also investigated the impact of AFB1 on PLC/PRF/5 cells on the transcriptome. The RNA-seq data revealed that in response to AFB1, 1048 genes were downregulated while 1445 genes were upregulated (Fig. 4a and Supplementary Table S4). Interestingly, multiple components of the P450-related metabolic pathway were identified among the group of significantly upregulated genes (Fig. 4b). Furthermore, on the basis of KEGG pathway analysis, the differentially expressed genes fell into two different functional clusters, with the upregulated genes associated with axon guidance in the development and the downregulated genes associated with cancer (Fig. 4c, d).

Importantly, AHR mRNA and proteins levels were significantly increased upon exposure to AFB1 (Fig. 4e, f). However, AFB1-induced expression of *CYP1A1* and *CYP1A2* mRNAs, encoding two major P450 family members, was suppressed in *AHR* deficient PLC/PRF/5 cells (Fig. 4g). *AHR* deficient HuH7 cells yielded similar results (Fig. 4h–j). The CYP1A1 and CYP1A2 expressions were evaluated of in AHR OE cells under AFB1 treatment. In both parental and OE cell populations, CYP1A1 and CYP1A2 were induced by AFB1 to similar levels (Supplementary Fig. S4a–c). Thus, AHR appeared to promote the expression of a specific subset of P450 metabolic enzymes that are likely to be associated with the metabolism of AFB1 as a xenobiotic substance and therefore can be considered as a therapeutic target for preventing the early development of aflatoxin-associated HCCs.

### AFB1 induces the nuclear translocation of AHR

**Fig. 5:**
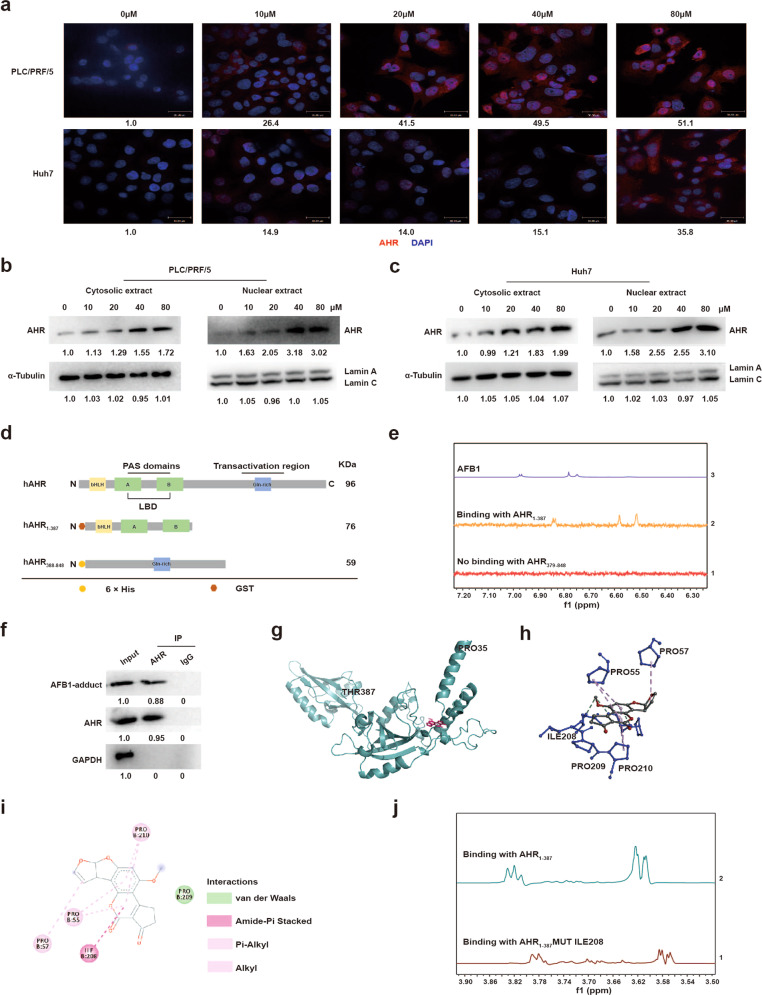
AFB1 activates AHR nuclear translocation and the N-terminus domain of AHR binds to AFB1. **a** Images of immunofluorescence staining to localize AHR (red) in PLC/PRF/5 and HuH7 cells with AFB1 treatment. DAPI (blue) was used to stain the nucleus. **b**, **c** Western blot analysis of AHR in **b** PLC/PRF/5 and **c** HuH7 cells. Cytosolic extracts are analyzed in panels on the left; nuclear extracts in panels on the right. **d** Domain structures of AHR N- and C-terminal recombinant proteins. **e** STD analysis of the AHR recombinant proteins bound to 20 µM AFB1. **f** Western blot analysis of co-IPs to assess the binding between AHR and AFB1. **g** The de novo modeling of AHR_1–387._ **h** The binding model of AFB1 on the molecular surface of AHR_1–387._ **i** The interaction model of AHR_1-387_ with AFB1. **j** STD analysis of the *AHR*_*1*–387_-Mut-ILE280 recombinant proteins with 20 µM AFB1

The dynamic subcellular partitioning of AHR is crucial for its function. Upon binding to its ligand, typically planar aromatic hydrocarbons, AHR shuttles from the cytoplasm to the nucleus and forms a heterodimer with the protein aryl hydrocarbon receptor nuclear translocator (ARNT)^25^. We, therefore, detected the cellular localization of AHR in treated cells by immunostaining and the results showed that AHR was specifically translocated to the nucleus upon exposure to AFB1 (Fig. 5a). The differential distribution of AHR was confirmed on a western blot with nuclear and cytoplasmic fractions prepared from either PLC/PRF/5 or HuH7 cells (Fig. 5b, c), suggesting that AFB1 may promote AHR activity in a manner similar to established AHR ligands.

STD analysis indicated that AFB1 interacted with AHR at the N-terminus within amino acids 1–387 (Fig. 5d, e and Supplementary Fig. S5a, b). Co-IPs confirmed the binding between AFB1 and AHR (Fig. 5f). Molecular insight into this binding was explored through de novo modeling (Fig. 5g, h). The structure model generated was statistically reliable with a C-score of −1.18. Docking simulation gave rise to the binding model of AFB1 with AHR_1–387_, with the highest affinity score of −7.5 (kcal/mol). Amide-pi stack and alkyl were features of the main binding region in AHR for AFB1, which involved residues P55, P57, I208, P209, and P210 (Fig. 5i). I208 was suggested as the key residue mediating the binding to AFB1, and a mutation of this residue, Mut-I280, severely disrupted the binding to AFB1 (Fig. 5j and Supplementary Fig. S5c). Finally, we reintroduced WT AHR or mutant AHR into *AHR* KD cells and assessed cell proliferation with AFB1 treatment. We found that the reexpression of WT AHR restored the sensitivity of liver cells to AFB1, while the reexpression of mutant AHR did not (Supplementary Fig. S5d, e).

### Expression of AHR is elevated in response to AFB1 in primary tumor samples

**Fig. 6:**
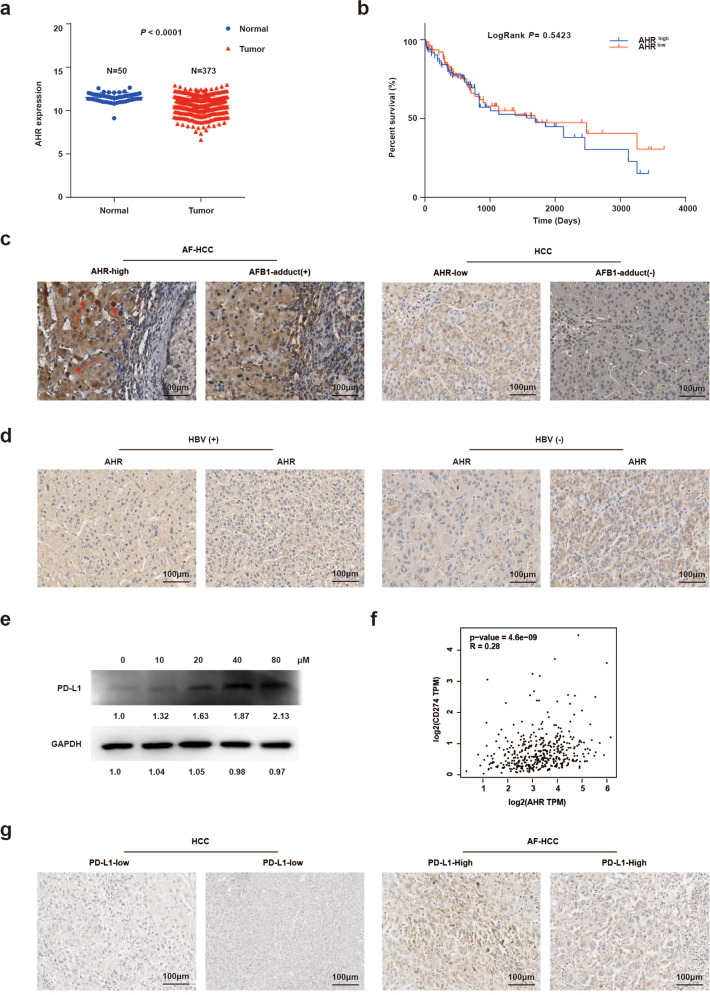
AHR and PD-L1 are highly expressed in primary AFB1-associated hepatocellular carcinomas (AF-HCC). **a** *AHR* expression levels in HCC using the TCGA dataset (*n* = 424)**. b** Kaplan–Meier plot for overall survival of hepatocellular carcinoma patients based on *AHR* expression levels in tumors from the TCGA dataset. High and low expression is based on the median level of *AHR* expression in all patients. **c**, **d** Images of immunohistochemical staining for AHR expression in hepatocellular tumors **c** with or without AFB1 exposure and **d** with or without HBV infection. **e** Western blot staining of PD-L1 in PLC/PRF/5 with increasing AFB1 concentrations treatment. **f** *AHR* and *PD-L1* expression correlation analysis using data from the GEPIA server. **g** Images of immunohistochemical staining for PD-L1 expression in hepatocellular tumors with or without AFB1 exposure

We next further explored the clinical relevance of AFB1-induced AHR upregulation by investigating the relationship between the levels of AHR and HCC development. First, we analyzed *AHR* mRNA levels in the array data from 424 liver cancers in the TCGA database (Fig. 6a). Differential expression of *AHR* was primarily due to the levels in nonneoplastic liver samples, contradictory to previous findings^26^. Furthermore, AHR expression levels were not associated with patient survival (Fig. 6b). Thus, we considered the possibility that other factors, including AFB1 exposure and HBV infection, might also affect *AHR* expression. We, therefore, evaluated AHR protein levels in a cohort of documented aflatoxin-associated liver cancers (AF-HCC) from patients with detectable levels of Aflatoxin M1 in their urine samples (Table 1 and Supplementary Table S5). IHC staining revealed higher levels of AHR in the AF-HCCs than in HCCs negative for AFB1 adducts. These AF-HCC samples also exhibited AHR nuclear translocation (Fig. 6c). However, no significant increase in AHR expression was found in the HBV-positive tumors (Fig. 6d). Taken together, these results indicated a possible link between AF-HCC and the AHR levels.

**Table 1:** *AHR* staining and clinicopathological characteristics of hepatocellular carcinoma patients

Variable	Number	*AHR* high staining	*AHR* low staining	*P* value
**Age No. (%)**				
>50	24(55.8)	13(65.0)	12(52.2)	0.3951
≤50	19(44.2)	7(36.0)	11(47.8)	
**Gender No. (%)**				
Male	31(72.1)	14(70.0)	17(73.9)	0.7754
Female	12(27.9)	6(30.0)	6(26.1)	
**Edmondson-Steiner grade No. (%)**				
I	1(2.3)	0(0)	1(4.3)	0.3454
II/III	42(97.7)	20(100.0)	22(95.7)	
**HBsAg No. (%)**				
(+)	30(70.0)	18(90.0)	12(57.1)	0.0533
(-)	13(30.0)	2(10.0)	9(42.9)	
**AFM1 in urine No. (%)**				
Y	10(23.2)	7(35.0)	3(13.0)	0.089
N.D.	33(76.7)	13(65.0)	20(87.0)	
**Cirrhosis No. (%)**				
Y	26(60.5%)	10(50.0)	16(76.2)	0.0818
N	17(39.5%)	10(50.0)	5(23.8)	

In our previous work, we found enhanced expression of PD-L1 in AF-HCC samples^8^. We found that AFB1 treatment also increased levels of PD-L1 in PLC/PRF/5 cells with the increasing AFB1 concentration (Fig. 6e). These results suggested that PD-L1 might function as one of the key downstream effectors of AFB1 exposure. Analysis of the TCGA data also suggested a close association between *AHR* and *PD-L1* expression (*R* = 0.28; *P* value = 4.6e-09 (Fig. 6f). Finally, in our cohort of AF-HCC samples, immunostaining demonstrated that increased AHR expression correlated with the upregulation of PD-L1 (Fig. 6g). Collectively, these data suggested that the signal and functional axis of AFB1-AHR were associated with the development of HCC.

### AHR activity improves anti-*PD-L1* therapy

**Fig. 7:**
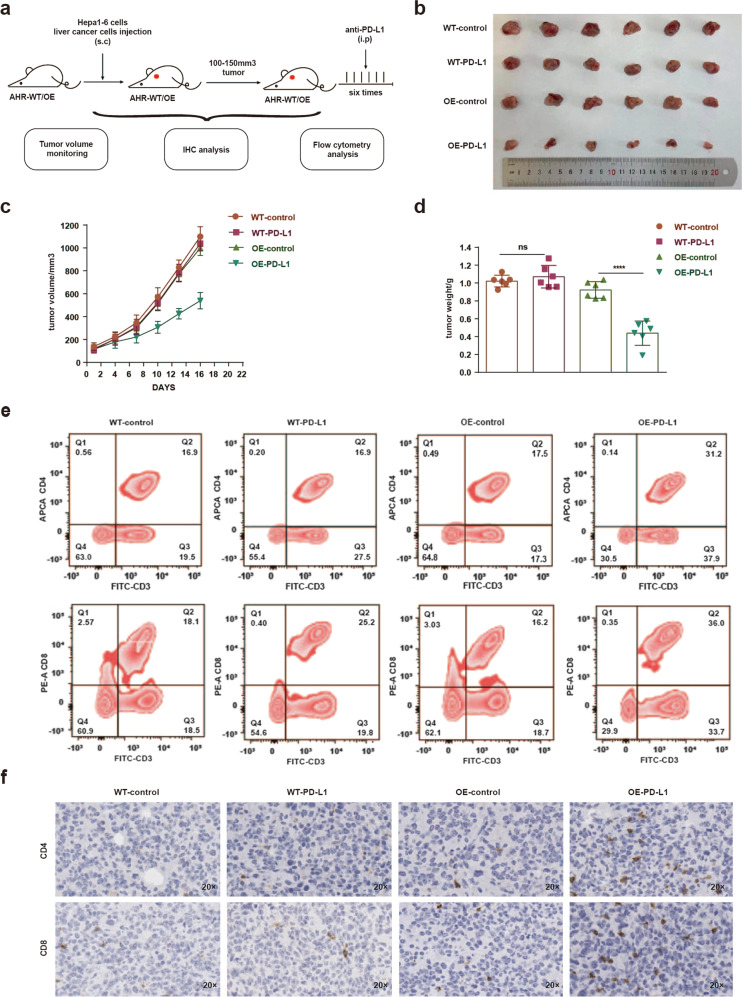
Increased efficacy of anti-PD-L1 therapy on liver cancer xenografts overexpressing AHR. **a** Schematic of the cycles anti-PD-L1 treatment of mice bearing liver tumor. **b** Representative images of xenograft tumors with anti-PD-L1 treatment. **c** Xenograft tumor volume plotted over days during anti-PD-L1 treatment. **d** Weight of xenograft tumors (*n* = 6) harvested from animals after anti-PD-L1 treatment. **e** Flow cytometry assessing the proportion of CD4^+^/CD8^+^ cells with fluorescent antibodies against the cell markers indicated. **f** Immunohistochemical staining for CD4/8 in sections from xenograft tumors with or without anti-PD-L1 treatment

To evaluate the impact of AHR expression on the efficacy of anti-PD-L1 therapy for HCC, we generated a xenograft model with hepa1–6 cells harboring a construct expressing *AHR*. Control xenografts were derived with cells transduced by the backbone vector. The anti-PD-L1 regimen of i.p. administration of blocking antibodies at 100 μg, every 4 days for six cycles, was initiated once the tumor volume exceeded 100 mm^3^ (Fig. 7a). Treatment significantly reduced the size of xenografts derived from *AHR*-OE cells relative to WT and Vector control xenografts (Fig. 7b). Both the volume and the weight of the xenografts paralleled these results (Fig. 7c, d). More importantly, we found a significantly less severe level of T cell exhaustion in *AHR*-OE xenografts. The fractions of proliferating CD4^+^/CD8^+^ T cells were at 31.4% and 36% in the *AHR*-OE xenografts, as opposed to 16.9% and 25.2% in the WT control tumors (Fig. 7e). These results were consistent with immunohistochemistry for CD4/8 which showed an increased number of CD4^+^/CD8^+^ T cells in *AHR*-OE tumors relative to controls (Fig. 7f). Finally, the results exhibited that PD-L1 expression was elevated in the xenografts with the AHR overexpression (Supplementary Fig. S6). Therefore, enhanced expression of AHR sensitized the tumors to anti-PD-L1 therapy.

## Discussion

As a genotoxic hepatocarcinogen, AFB1 is metabolized by cytochrome P450 metabolizing enzymes into AFB1-8, 9 epoxide (AFBO) which is reactive and forms adducts with DNA. Dietary aflatoxins and hepatitis virus infection account for two of the major risk causes for the progress of primary HCC, which is one of the most common malignancies worldwide. The relatively higher occurring frequencies of these two factors have raised a significant public health concern for the development of AFB1-associated liver cancer in some regions of China. However, beyond the established genotoxicity of AFB1, the mode of action remains largely unexplored. Excessive accumulation of LCFAs acids is one of the ways AFB1 exerts toxicity and LCFAs do accumulate during necroptosis and decreased cell proliferation^24^. In this study, through a comprehensive loss-of-function genetic screen, we have identified AHR as another crucial mediator for AFB1-induced cellular toxicity.

AHR was first discovered based on its high affinity for TCDD. Since then, the chemical spectrum of AHR ligands has been greatly expanded from naturally occurring tryptophan derivatives, bacterial metabolites, and polyphenols to artificial halogenated and polycyclic aromatic hydrocarbons^27,28^. The biological validation of AHR ligands relies on monitoring the activation of the downstream target genes such as *CYP1A1* and *CYP1A2* or the reporter genes derived from the endogenous targets. We found that the increase in the levels of *CYP1A1* and *CYP2A2* induced by AFB1 requires AHR, further supporting the notion that AFB1 is a potential AHR ligand. The STD analysis showed that AFB1 indeed binds directly to AHR. Furthermore, in vitro binding experiments identified a binding site for AFB1 in the N-terminus (AA1-387) of AHR and I280 as the critical residue mediating the interaction.

The level of AHR is reportedly significantly elevated in various forms of cancer, including breast, ovarian, and liver cancers, and the AHR target genes of *CYP1A1* and *CYP1A2* are also linked to prognosis^29–31^. Despite the poor correlation between *AHR* expression and liver cancer occurrence based on the TCGA data, our work revealed that AHR protein levels increase in response to AFB1-adduct formation, highlighting its importance in studying aflatoxin-related liver cancer.

AHR plays a central role in the development of BαP-induced lung cancer^32^. Differential AHR levels are commonly found in the comparison of tumor samples from smoking and nonsmoking patients^33^. Based on the dynamic nature of AHR levels revealed in this work, especially in response to AFB1-related stimuli, we propose the use of AHR as a monitor for the risk in developing liver cancer.

Moreover, anti-PD-L1 antibody therapy has been given to non-small cell lung carcinoma (NSCLC) patients. PD-L1, a molecule involved in cancer immune evasion, is also found frequently upregulated in the samples derived from smoking patients. AHR can be directly bound to the region of the PD-L1 promoter and induce its expression. This result is supported by the fact that higher AHR levels were also found in 81.3% of the NSCLC tumors from patients chosen to receive anti-PD-L1 antibody therapy based on high levels of PD-L1^34^. Our results also showed a close correlation between the levels of AHR and PD-L1. More importantly, the enhanced expression of AHR sensitizes the tumors to anti-PD-L1 therapy. We, therefore, propose using AHR as a marker for the use of PD-L1-based immunotherapy in the treatment of patients with AF-HCC. In addition, in immunotherapy for the treatment of ovarian epithelial cancer, AHR has been proposed as a crucial factor for successfully targeting the pathways dependent on MyD88 and IDO1^30^. We, therefore, suggest that the combination of anti-IDO1 and anti-PD-L1 treatments might improve the efficacy of therapy against cancers that tend to evade single immunosuppressive treatment^35,36^.

The connection between AHR and AFB1-HCC, addressed by this work, is particularly relevant to the current clinical practices in combating HCC, especially when considering that the incidence of liver cancer has remained alarmingly high in China with limited options for effective therapies. Aflatoxin-associated HCCs have a characteristic high mutation rate dominated by C/A mutants, which often results in a significant increase in the development of mutation-associated neoantigens (MANAs). Importantly, the increased mutational burden is closely associated with the sensitivity to anti-PD-1/PD-L1 therapy^8,37^. We hope this work inspires the exploration of the potential of immunotherapy for AFB1-HCC via targeting AHR-regulated pathways.

## Materials and methods

### Ethics statement

The study was approved by the Human Research Ethics Committee of Hunan Cancer Hospital and the Ethics Committee of Cancer Hospital Chinese Academy of Medical Science.

### Sample collection

Paired aflatoxin-associated liver tumors and adjacent noncancerous tissues (*n* = 10 pairs) were collected in the Qidong Liver Cancer Hospital Institute between 1993 and 1998. Additional HCC tissues (*n* = 33) were obtained from Cancer Hospital Chinese Academy of Medical Science, Beijing, China. Clinicopathological characteristics of patients are summarized in Table S5.

### Cell culture

The PLC/PRF/5 and L-O2 cell lines were purchased from the National Infrastructure of Cell Line Resource (Beijing, China). The HuH7 cell line was purchased from the Cell Bank of the Chinese Academy of Sciences (Shanghai, China). PLC/PRF/5 and HuH7 cell lines were cultured in DMEM. The L-O2 cell line was cultured in RPMI-1640. All culture medium supplemented with 10% FBS and penicillin (100 U/mL)/streptomycin (0.1 mg/mL).

### Screening CRISPR libraries with AFB1

Brunello CRISPR knockout pooled library infected PLC/PRF/5 cells with 500-fold genome-wide coverage and at an MOI of ~0.3. After infection for 24 h, puromycin treatment for 48 h to obtain successfully infected cells. Transduced cells (3.822 × 10^8^) were treated with 8 µM AFB1 for 48 h. Under AFB1 treatment, ~90% of the cells rounded up and died. When the remaining cells reached 30–40% confluence, a fresh medium containing 8 µM AFB1 was added to cells. After six rounds of AFB1 treatment, 4.01 × 10^5^ cells were collected for genomic DNA isolation to identify sgRNAs.

### Individual sgRNA lentivirus production and infection

The lentiCRISPR v2 system was used to generate lentiviruses containing individual sgRNAs for validation. The day before transfection, HEK293T cells (4 × 10^7^ per six-well) were seeded into six-well plates to achieve at least 70% confluence. HEK293T cells were transfected with lentiCRISPR v2, psPAX2 (#12260, Addgene), and pMD2.G (#12259, Addgene) plasmids using Neofect transfection reagent (Neo Biotech, Beijing, China). The supernatant was collected after 60 h, centrifuged to remove particulate matter, and filtered through a PVDF filter membrane (0.45 µM; Millipore; Shanghai, China).

### Quantitative real-time PCR

Total RNA was extracted with TRIzol reagent (Thermo Fisher Scientific, Waltham, MA, USA), and the PrimeScript RT Reagent Kit (TaKaRa; Tokyo, Japan) RNA (500 ng) was used to reverse transcribe RNA into cDNA. RT-PCR was conducted with the SYBR^®^ Premix Ex Taq™ on the ABI V7 (ABI; Indianapolis, IN, USA). The primers sequences used are shown in Table S6.

### SiRNA transfection

Cells (3 × 10^5^) were plated into six-well plates and transfected with siRNAs (GenePharma, Shanghai, China) using RNAimax (Life Technologies; Brendale QLD, Australia). The final concentration of siRNA is 20 µM. Cells were collected 24 h after transfection. Sequences of the siRNAs are shown in Table S7.

### Western blot analysis

The total protein was extracted from cells with lysis buffer (1 M Tris-HCL (PH 6.8), 80% glycerin, and 10% SDS. The concentration of the extracted protein was then determined by the BCA kit (Beyotime Biotech, Nantong, China). Protein lysates (30 μg) were separated using SDS-PAGE gel and electro-transferred onto a PVDF membrane (Millipore). Membranes were blocked with 5% milk in TBST and incubated with primary antibodies overnight at 4 °C. HRP-conjugated secondary antibodies were incubated for visualization and quantification of proteins. Antibodies used to determine protein expression were the following: AHR (#83200 S; Cell Signaling Technology, Danvers, MA, USA), AFB1-adduct (#NB600-443; Novus Biologicals, Centennial, CO, USA), CYP1A1 (#13241-1-AP; Proteintech, Rosemont, IL, USA), CYP1A2 (#19936-1-AP; Proteintech), and GAPDH (#60004-1-AP; Proteintech), α-tubulin (#11224-1-AP; Proteintech), lamin A/C (#4777; Cell Signaling Technology, Danvers, MA, USA), and PD-L1(#13684 T; Cell Signaling Technology).

### Clone formation assay

Cells (1 × 10^4^) were inoculated in six-well plates. After incubation for another 10 days, 4% paraformaldehyde was added for fixation of surviving colonies, which were then stained with 0.1% crystal violet.

### Co-immunoprecipitation

Transfected cells were collected in lysis buffer (#C1050, Applygen Technologies; Beijing, China) with Phosphatase Inhibitor Cocktail II (#HY-K0022, MedChemExpress; Monmouth Junction, NJ, USA) and Protease Inhibitor Cocktail (#HY-K0010, MedChemExpress) at 4 °C, and lysates were centrifuged at 12,000x*g* for 15 min. Supernatants were incubated first with IgG antibody, subsequently with beads for 1 h, and finally primary antibodies overnight. The immunocomplexes were rinsed several times with PBST, and the precipitated beads were resuspended for electrophoresis. The primary antibodies used were the following: AHR (#83200 S; Cell Signaling Technology), AFB1-adduct (#NB600-443; Novus Biologicals), and IgG (#2729 S, #5415 S, Cell Signaling Technology,).

### RNA-seq

RNA-seq was performed by Majorbio company (Beijing, China). RNA-seq library was prepared with a TruSeqTM RNA sample preparation kit (Illumina; San Diego, CA, USA). The original paired-end readings are trimmed and quality controlled using SeqPrep (https://github.com/jstjohn/SeqPrep) and Sickle (https://github.com/najoshi/sickle). TopHat software was used to align the clean sequencing reads to the human genome (hg38). RSEM was used to quantify gene abundance and EdgeR was used for differential expression analysis^38,39^. Gene Ontology and KEGG pathway analysis were performed using KOBAS2.1.1(http://kobas.cbi.pku.edu.cn/download.php).

### Determination of AFB1 sensitivity

IC50 values determined in the CCK8 assay were used to assess the sensitivity of liver cancer to AFB1 toxicity. In brief, the cells (PLC/PRF/5 and HuH7 cells) were plated in 96-well plates at a density of 4 × 10^3^ cells/well. The cells were supplemented in a medium containing different concentrations of AFB1, the Cell Counting Kit-8 (DOJINDO; Kumamoto, Japan) was used to measure the cell viability.

### Immunohistochemistry and immunofluorescence

Cells were seeded onto microscope slide cover glass in 12-well plates and treated with different concentrations of AFB1 and then removed from the medium followed by 20 min fixation with 4% paraformaldehyde. Following three rinses, the cells were permeated with 0.5% Triton X-100 in PBS for 10 min, treated with 3% BSA for 30 min, and subject to overnight incubation with primary antibody at 4 °C. Cells were rinsed with PBS and incubated with Alexa Fluor^®^ 647 conjugated antibody or biotinylated secondary antibodies for at least 1 h. Staining regimen with DAPI (1 μg/mL, Sigma-Aldrich; St. Louis, MO, USA) for nuclear labeling. The LCFAs were detected with Nile Red staining. Images were captured under laser confocal microscopy. The coloration of IHC was performed using the DAB Kit (ZSGB-Bio; Beijing, China).

### Nontargeted metabolomics profiling

PLC/PRF/5 cells were plated in 100 mm dishes with DMSO or AFB1 (20 µM) treatment for 48 h. Extraction and analysis of the metabolites were performed as previously described^40^. The selection criteria of the differential metabolites were the ratios of the *AHR*-WT-AFB1 group to the *AHR*-WT-DMSO of >1.5 or <0.75. Metaboanalyst 4.0 was used to perform the pathway analysis.

### AHR recombinant proteins

The sequence for *AHR*_1–387_ and *AHR*_1–387_-Mut-ILE280 were inserted into the pGEX-6p-1 vector to construct a GST-tagged protein, and *AHR*_388–848_ was inserted into the PET28A vector to construct a recombinant protein with His6-tag. The constructs were isolated from selected clones using the Endo-Free Plasmid Mini Kit II (D6950; Omega Bio-tek; Norcross, GA, USA), confirmed with Sanger sequencing, and transformed into *E. coli* BL21 (DE3) for expression. Bacteria containing confirmed constructs were inoculated in 250 mL of LB medium, and when the OD_600_ reached 0.8, 1 mmol/L IPTG was added to induce protein expression. Bacteria were collected after 6 h, resuspended in PBS with Protease Inhibitor Cocktail (CWBIO), and lysed by sonication on ice. The lysate was purified with GSTrap HP columns, and eluted with Tris-HCL (50 mM) and reduced glutathione (10 mM). AHR_388–848_ was purified by Hangzhou HuaAn Biotechnology Company (Hangzhou, China). Recombinant proteins were validated on SDS-PAGE.

### Saturation-transfer difference (STD) NMR

The AHR recombinant protein was dialyzed to remove Tris. For AFB1, 5 mM stock solutions in deuterated DMSO (DMSO-d6) were prepared. Five microliters of AFB1 (20 µM) with 20 µL of D_2_O were added to the protein for NMR analysis. The pulse program stddiffgp 19.3 was performed for the STD experiment. The saturation time and shape pulse power for saturation were set to be 2 s and 40 dB, respectively.

### De novo modeling and molecular docking

ChemDraw was used to draw the planar conformation of the ligand molecule AFB1, the 3D conformation was formed in Chem3D, and the energy was optimized under the MM2 force field. Semiflexible docking was chosen, with a flexible ligand small molecule conformation, and a rigid receptor protein conformation. Search scope was established with a gridbox with the center of (29.557: 27.176: −32.974) and a size of (56:52:74), generating possible binding conformations (num_modes = 20). PYMOL software was used to remove excess protein structures, water molecules, and other unrelated ligands of AHR_1–387_. Molecular docking was realized with the AutoDock vina program, and conformation search strategy, with the quasi-Newton algorithm^41^.

### Flow cytometry

Whole blood (50 µL) was obtained from mouse orbit and added to an anticoagulant tube containing heparin. Add RBC lysate (2 mL) to each tube and lysate were centrifuged at 1000 rpm for 5 min. All staining buffer and washes were performed in freshly prepared staining buffer (PBS containing 1% FBS). Staining of murine CD4/8^+^ T cells was performed with the following antibodies: anti-mouse FITC-CD3 (#552062, clone:145-2C11; BD Pharmingen), PE-cy7-CD45 (CD451020419603, clone:30-F11; TONBO Biosciences), APC-CD4 (#553051, clone:RM4-5; BD Pharmingen,) and PE-CD8 (#553033, clone:53-6.7; BD Pharmingen). The tubes were incubated for 20 min away from light. Data were acquired using an LSR-II (Becton Dickinson; Franklin Lakes, NJ, USA) and analyzed using Flow Jo software (Tree Star Inc.).

### Mice and anti-PD-L1 treatment

C57BL/6 mice were purchased from Beijing Huafukang Biological Technology Co., Ltd. (Beijing, China). About 1 × 10^6^ Hepa1–6 cells were subcutaneously injected into each nude mouse randomly. The administration group was subjected to intraperitoneal injection with PD-L1 antibodies (clone:10 F.9G2), every 4 days, with a total of six cycles starting when the tumor volume reached about 100 mm^3^. Blood was taken before and after anti-PD-L1 treatment to characterize immune cell types. Tumor volume was measured manually after every anti-PD-L1 treatment and the total volume was calculated as (a × b^2^)/2 (a = longest length of diameter, b = shortest length in diameter).

### Statistical analysis

The statistical analysis was performed with GraphPad Prism 6.0 software and measurement data were summarized as the mean ± SD. The two-tailed *t*-test was adopted for the comparison of data between groups. For all analyses, *P* < 0.05 indicated that the difference was statistically significant.

## Supplementary information

Supplementary materials

Supplementary tables

## Data Availability

All data generated or analyzed during this study are included in this published article.
