# Correction: AHR mediates the Aflatoxin B1 toxicity associated with hepatocellular carcinoma

**DOI:** 10.1038/s41392-021-00794-y

**Published:** 2021-12-20

**Authors:** Qing Zhu, Yarui Ma, Junbo Liang, Zhewen Wei, Mo Li, Ying Zhang, Mei Liu, Huan He, Chunfeng Qu, Jianqiang Cai, Xiaobing Wang, Yixin Zeng, Yuchen Jiao

**Affiliations:** 1 State Key Laboratory of Molecular Oncology, National Cancer Center/National Clinical Research Center for Cancer/Cancer Hospital, Chinese Academy of Medical Sciences and Peking Union Medical College, Beijing, China; 2 State Key Laboratory of Medical Molecular Biology, Institute of Basic Medical Sciences Chinese Academy of Medical Sciences, School of Basic Medicine Peking Union Medical College, Beijing, China; 3 Department of Hepatobiliary Surgery, National Cancer Center/National Clinical Research Center for Cancer/Cancer Hospital, Chinese Academy of Medical Sciences and Peking Union Medical College, Beijing, China; 4 Key Laboratory of Gene Editing Screening and R&D of Digestive System Tumor Drugs, Chinese Academy of Medical Sciences, Peking Union Medical College, Beijing, China; 5 State Key Laboratory of Oncology in South China, Collaborative Innovation Center for Cancer Medicine, Sun Yat-sen University Cancer Center, Guangzhou, China; 6 Department of Clinical Laboratory, National Cancer Center/National Clinical Research Center for Cancer/Cancer Hospital, Chinese Academy of Medical Sciences and Peking Union Medical College, Beijing, China

**Keywords:** Gastrointestinal cancer, Gastrointestinal cancer

Correction to: *Signal Transduction and Targeted Therapy* 10.1038/s41392-021-00713-1, published online 9 August 2021

After online publication of the article^1^, the authors noticed one inadvertent mistake occurred during the production process in Fig. 7 that needs to be corrected. The correct data are provided as follows. The key findings of the article are not affected by these corrections. The original article has been corrected.

**Fig. 7:**
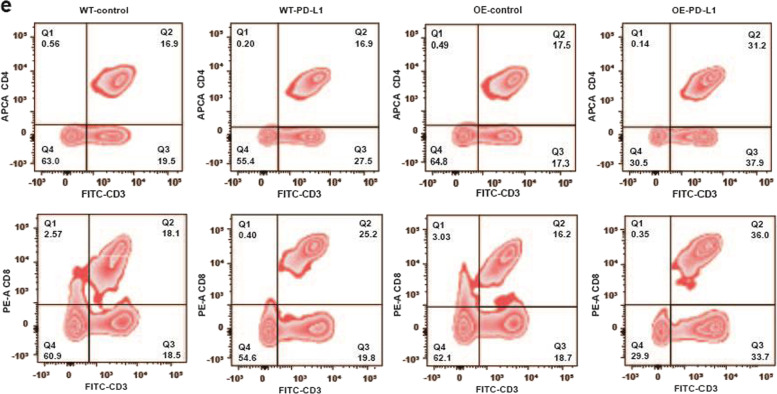
**e** Flow cytometry assessing the proportion of CD4/8+ cells with fluorescent antibodies against the cell markers indicated.
