# Supplementary material for: AHR mediates the aflatoxin B1 toxicity associated with hepatocellular carcinoma
Source: Signal Transduct Target Ther. 2021 Aug 9;6:299. doi: 10.1038/s41392-021-00713-1 (PMC8352983; doi:10.1038/s41392-021-00713-1)
Supplement: Supplementary file 1 — Supplementary materials [file 41392_2021_713_MOESM1_ESM.docx]

Supplementary Materials for

AHR mediates the Aflatoxin B1 toxicity associated with hepatocellular carcinoma

Qing Zhu^1,#^，Yarui Ma^1,#^, Junbo Liang^2,#^, Zhewen Wei^3,#^, Mo Li^1^, Ying Zhang^1^, Mei Liu^1^, Huan He^1^, Chunfeng Qu^1^, Jianqiang Cai^3^, Xiaobing Wang^1,4,*^, Yixin Zeng^1,5,*^, Yuchen Jiao^1,4,6,*^

Correspondence to: Yuchen Jiao(jiaoyuchen@cicams.ac.cn), Yixin Zeng(zengyx@sysucc.org.cn) and Xiaobing Wang(wangxb@cicams.ac.cn)

**This PDF file includes:**

Supplementary Figs. S1 to S6

**Other Supplementary Materials for this manuscript include the following:**

Supplementary Tables S1 to S7 [Excel document]

Supplementary Fig. S1


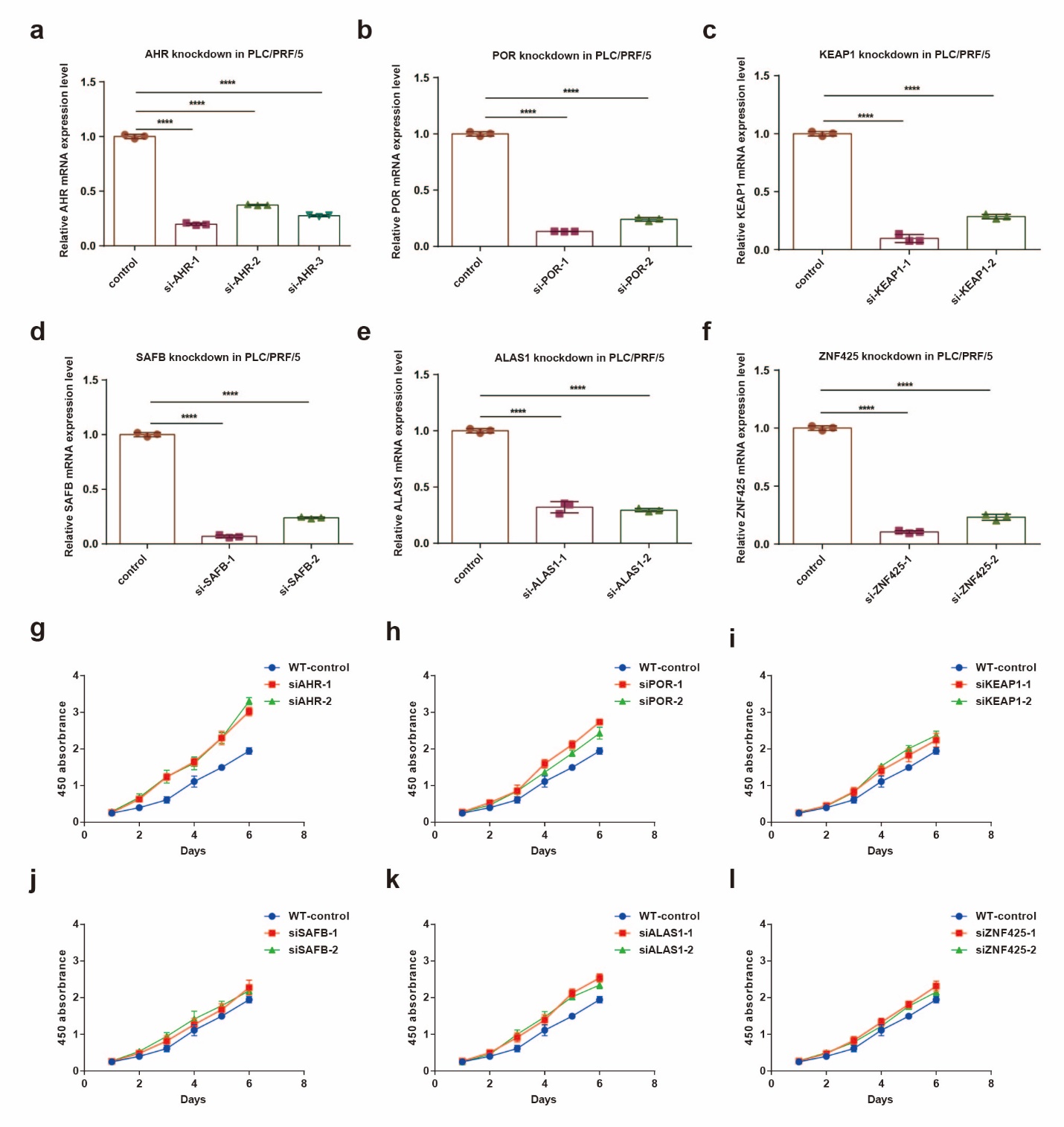


**Supplementary Fig. S1 Candidate resistance genes with AFB1 treatment.**

**a-f.** qPCR to verify the knockdown of **(a)** *AHR*, **(b)** *POR*, **(c)** *KEAP1*, **(d)** *SAFB*, **(e)** *ALAS1* and **(f)** *ZNF425*. **g-l**. Cell viability assessed with the CCK8 assay for HuH7 cells with knockdown of **(g)** *AHR*, **(h)** *POR*, **(i)** *KEAP1*, **(j)** *SAFB*, **(k)** *ALAS1* and **(i)** *ZNF425* under treatment with the 20 µM AFB1.

Supplementary Fig. S2


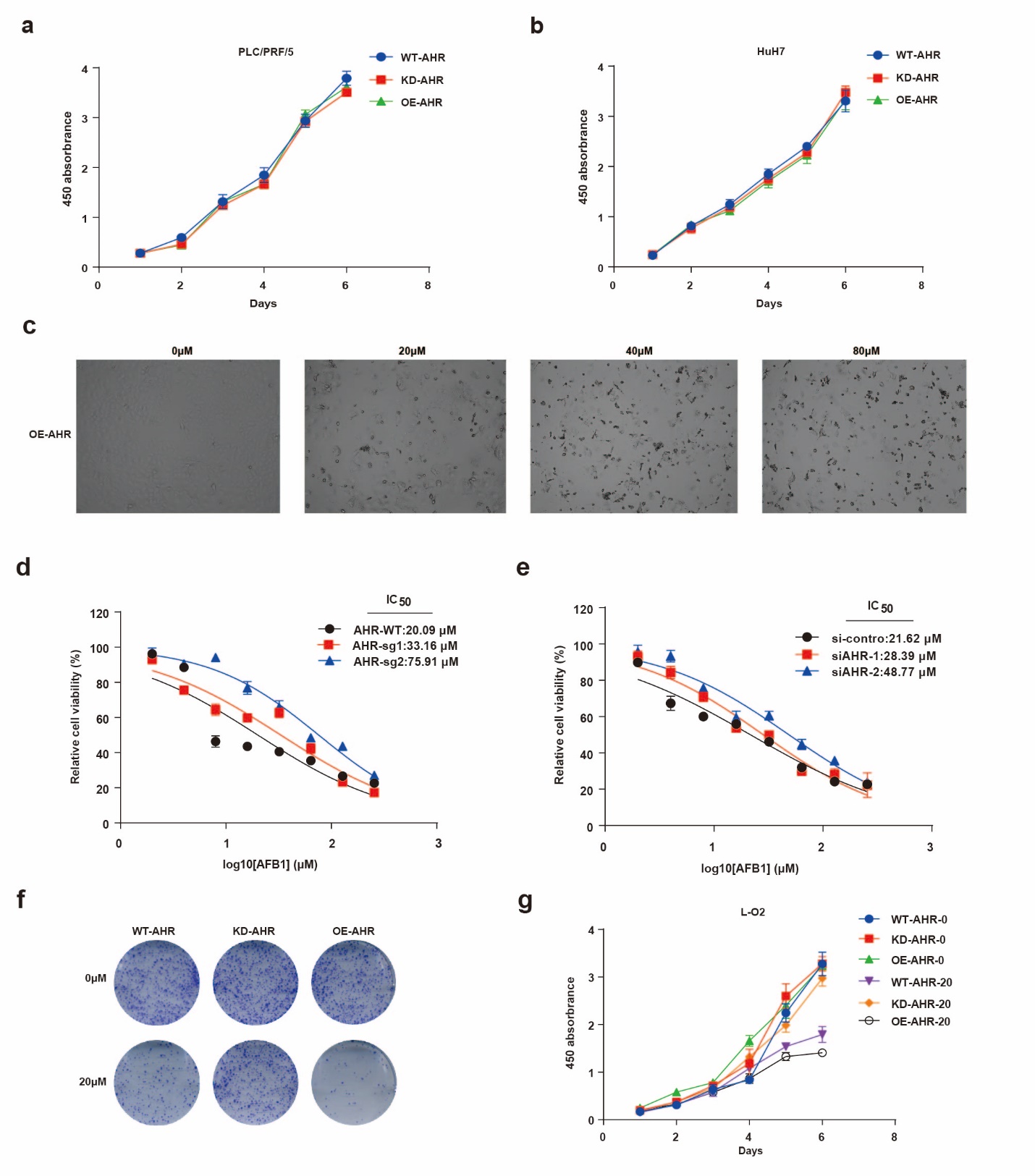


**Supplementary Fig. S2 *AHR* deficiency increases resistance of liver cancer cells to AFB1 treatment.**

**a, b.** Cell viability assessed with the CCK8 assay in **(a)** PLC/PRF/5 and **(b)** HuH7 cells with knockdown of *AHR* without AFB1 treatment. **c.** Representative images of AHR OE cells exposed to increasing concentrations of AFB1 for 48 hr. The images were taken using a LEICA inverted microscope (DMI 4000B). **d, e.** IC50 of AFB1 assessed with the CCK8 assay in *AHR* WT and *AHR* KD HuH7 cells treated with AFB1 for 48 hr. *AHR* knockdown was achieved using CRISPR sgRNA technology in **(d)** and siRNA in **(e)**. **f.** Images of colony forming assays for L-O2 cells with or without AFB1 treatment and stained with crystal violet. **g.** Cell viability assessed in L-O2 cells with or without AFB1 treatment with the CCK8 assay.

Supplementary Fig. S3


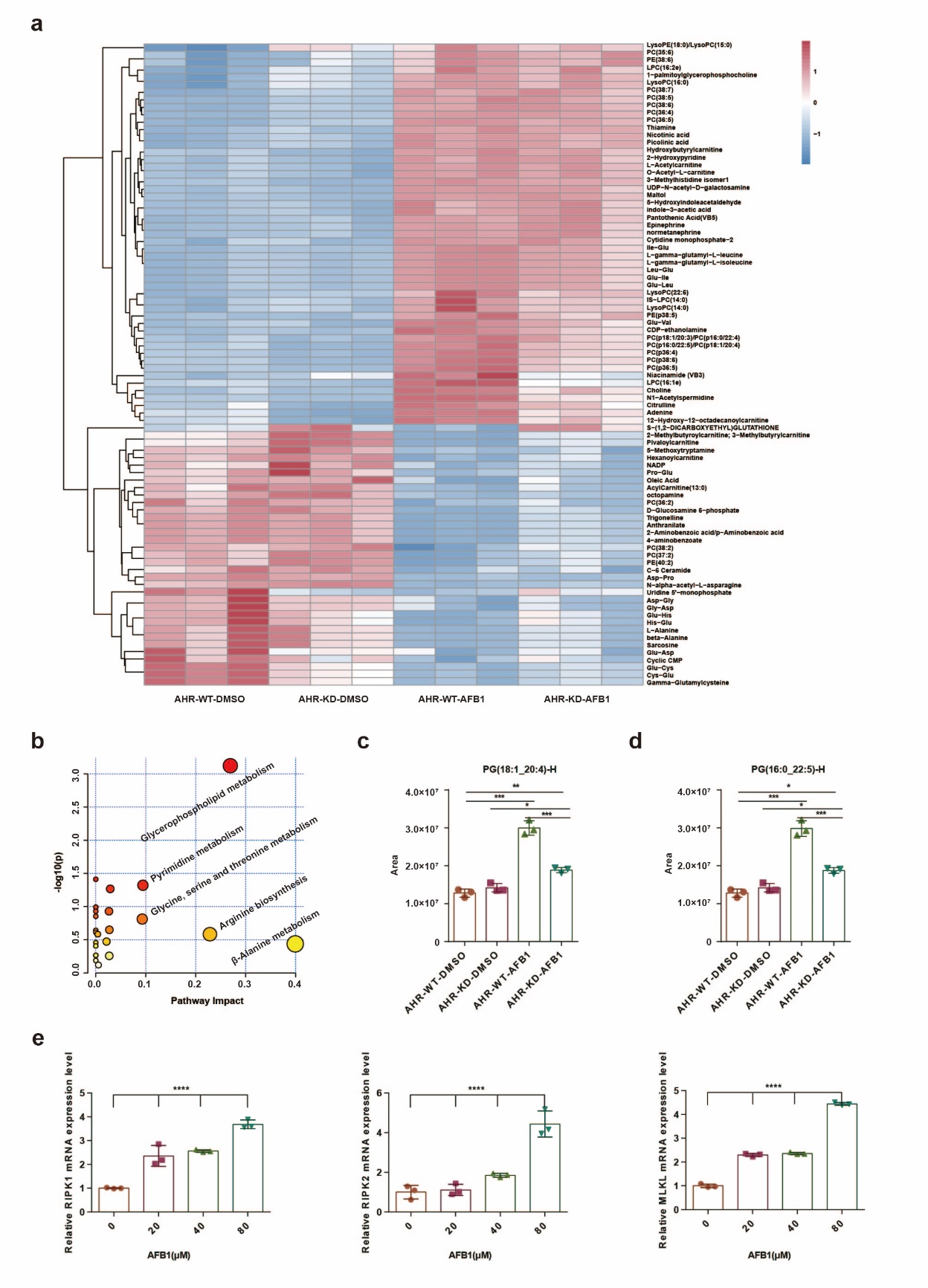


**Supplementary Fig. S3 The abundant metabolites** **in the positive (ESI+) ion mode.**

**a.** The heatmap of regulated metabolites extracted from the *AHR*-WT-DMSO, *AHR*-KD-DMSO, *AHR*-WT-AFB1, and *AHR*-KD-AFB1 groups in the positive ion mode. **b.** Pathway enrichment analysis of regulated metabolites of the four groups in the positive ion mode. **c, d.** The level of **(c)** PG(18:1_20:4)-H and **(d)** PG(16:0_22:5)-H was detected by LC–MS/MS. **e.** qPCR to determine *RIPK1*, *RIPK2* and *MLKL* mRNA expression in PLC/PRF/5 cells with AFB1 treatment. The results were expressed as mean ± SD. Data among multiple groups were analyzed using two-way ANOVA, * p < 0.05, ** p < 0.01, and *** p < 0.001.

Supplementary Fig. S4


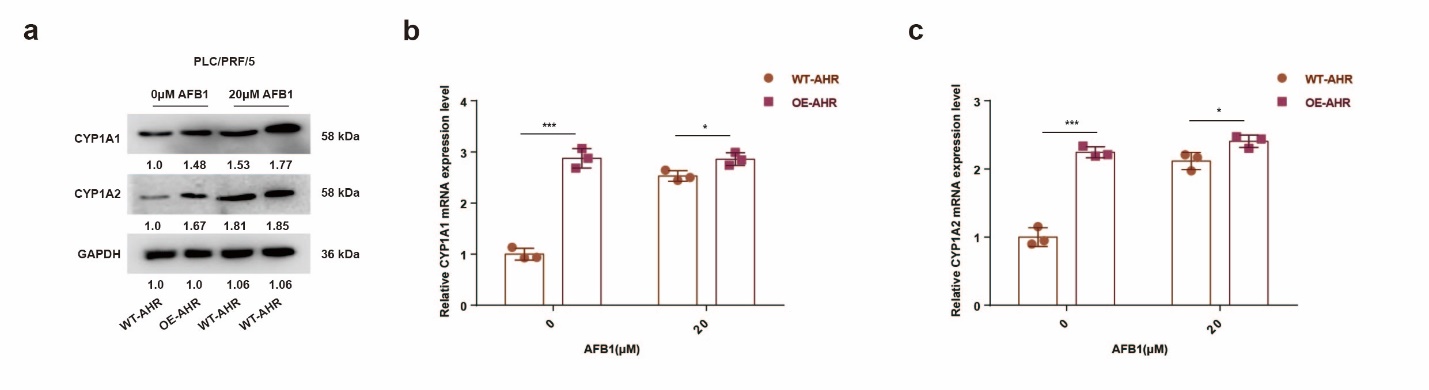


**Supplementary Fig. S4** **AFB1 induces the expression of CYP1A1 and CYP1A2*.***

**a.** Western blot analysis for CYP1A1 and CYP1A2 in WT AHR and OE AHR cells with or without AFB1 treatment. **b, c.** qPCR to determine **(b)** *CYP1A1* and (c) *CYP1A2* mRNA expression levels in WT AHR and OE AHR PLC/PRF/5 cells with or without AFB1 treatment.

Supplementary Fig. S5


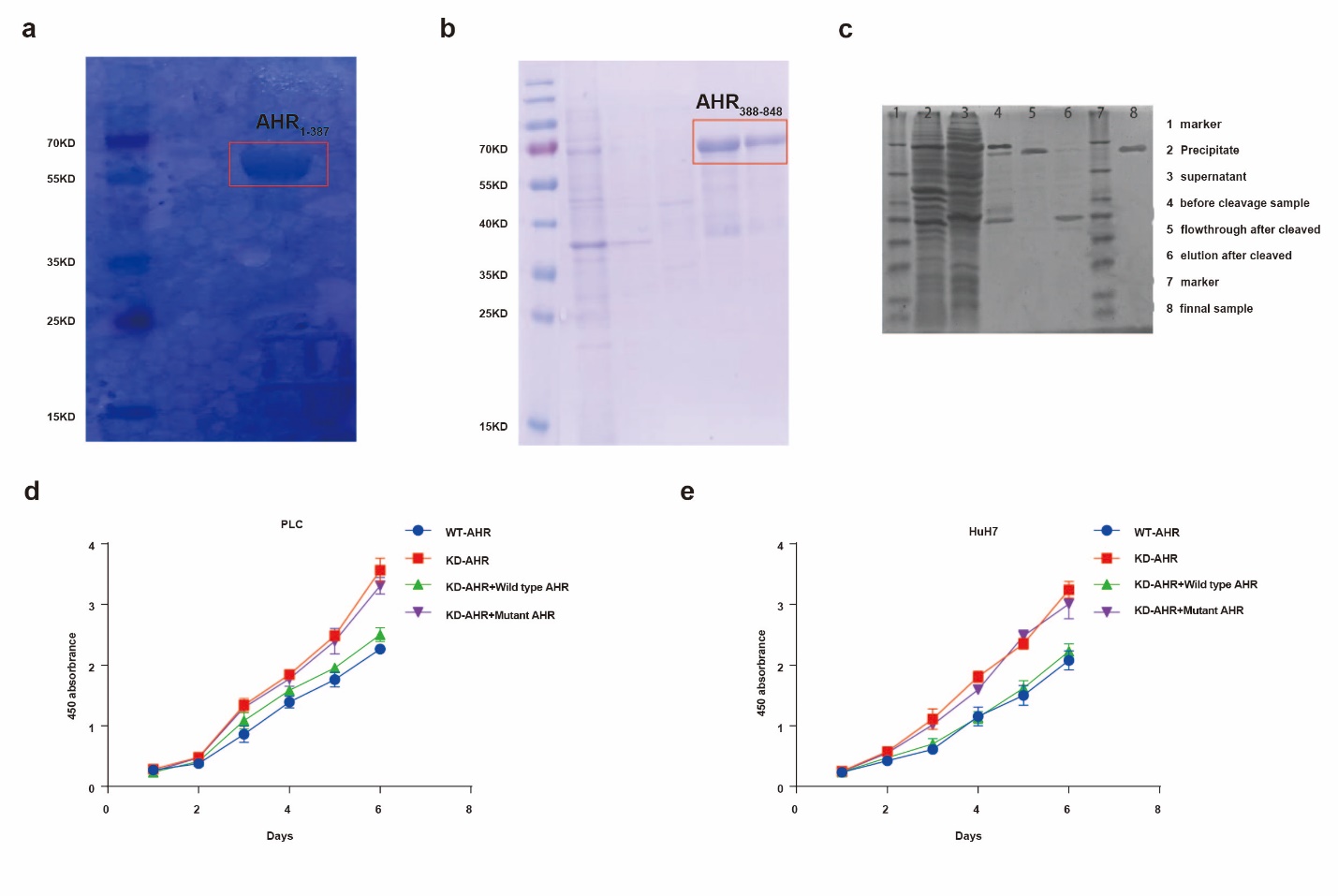


**Supplementary Fig. S5** **Purification of AHR mutant.**

**a, b.** SDS-PAGE for purified **(a)** AHR_1-387_ and **(b)**AHR_388-848_ and c. AHR1-387-Mut-ILE280 proteins. **d, e**. Cell viability assessed with the CCK8 assay in **(d)** WT AHR and OE AHR PLC/PRF/5 cells and **(e)** HuH7 cells with the re-expression of wild type AHR or mutant AHR with AFB1 treatment.

Supplementary Fig. S6


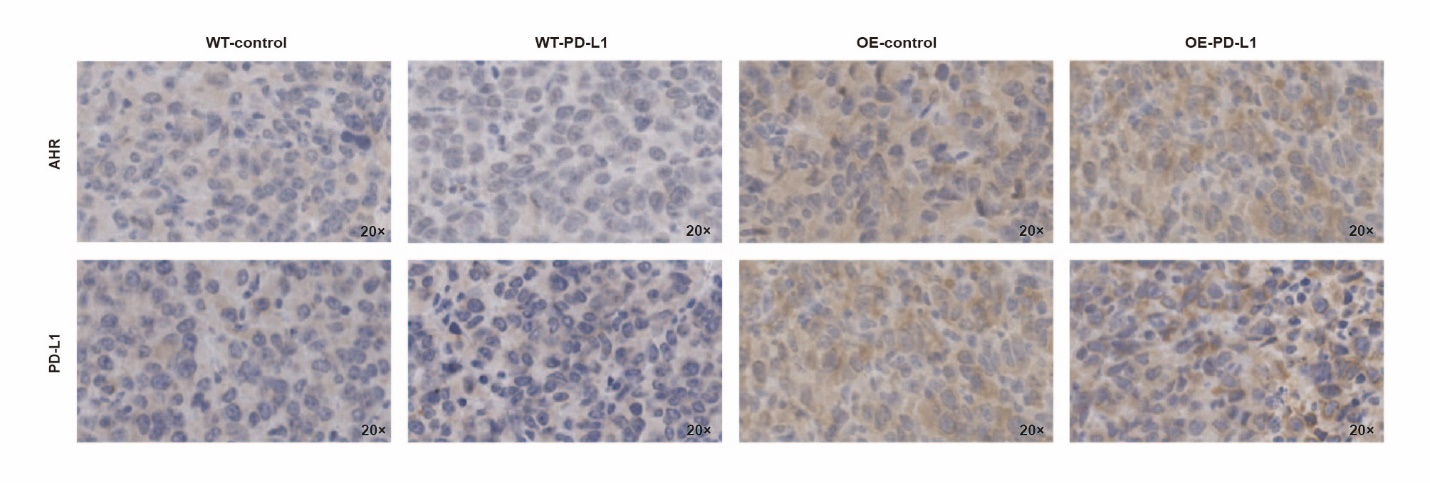


**Supplementary Fig. S6 AHR and PD-L1 staining in xenografts.**

Immunohistochemical staining for AHR and PD-L1 in xenograft tumors with or without anti-PD-L1 treatment.
